# Influence of sociodemographic factors and Family Health Strategy coverage on oral health promotion procedures: an analysis of Brazilian municipalities in 2019

**DOI:** 10.4317/jced.60404

**Published:** 2023-08-01

**Authors:** Suyene-de Oliveira Paredes, Edson-Hilan-Gomes de Lucena, Mauro-Henrique-Nogueira-Guimarães Abreu, Franklin-Delano-Soares Forte

**Affiliations:** 1 Post-graduation Program in Dentistry. Federal University of Paraíba, João Pessoa, Paraíba, Brazil; 2 Federal University of Minas Gerais, Belo Horizonte, Minas Gerais, Brazil

## Abstract

**Background:**

The aim of the study was to investigate associations between sociodemographic factors and municipal Family Health Strategy (FHS) coverage and oral health promotion (OHP) procedures in Brazil.

**Material and Methods:**

Data were obtained using public information systems and by direct request to the Ministry of Health. Clinical and collective OHP procedures performed in 2019 were analyzed, and sociodemographic covariates were associated with FHS coverage (population covered by FHS teams [FHST] and oral health teams [OHT]). Negative binomial regression models associated outcomes with covariates and estimated the prevalence ratio (PR) and confidence intervals (95%CI).

**Results:**

A total of 4,913 municipalities were included. Municipalities with low-income inequality (PR=1.04, 95%CI 1.01 to 1.08), high illiteracy rate (RP=1.06, 95%CI 1.00 to 1.13), and population size of 10,001 to 50,000 inhabitants (PR=1.07, 95%CI 1.02 to 1.12) and 50,001 to 100,000 (PR=1.21, 95%CI 1.12 to 1.30) showed a higher frequency of clinical procedures. In contrast, a low frequency of clinical procedures was associated with reduced vulnerability to poverty (PR=0.83, 95%CI 0.78 to 0.89) and low OHT coverage (PR=0.39, 95%CI 0.33 to 0.45). Regarding collective procedures, the final model showed associations between low frequency and reduced income inequality (PR=0.91, 95%CI 0.87 to 0.95), low per capita income (PR=0.84, 95%CI 0.81 to 0.88), and low (PR=0.53, 95%CI 0.35 to 0.80) and medium Human Development Index (PR=0.79, 95%CI 0.71 to 87).

**Conclusions:**

Clinical and collective OHP procedures were associated with sociodemographic conditions and OHT coverage in the FHS.

** Key words:**Health Promotion, Oral Health, Social Determinants of Health, Universal Health Coverage.

## Introduction

Health promotion allows people to improve health beyond individual behavior by increasing control over the determinants of health (1). This process transcends abilities and capacities of individuals and considers socioeconomic and environmental conditions (e.g., relationships among health, environmental, socioeconomic, and lifestyle conditions) to improve public health.

In Brazil, health promotion is contemplated in public health policies. The National Health Promotion Policy expresses the Federal, State, and Municipal intersectionality and co-responsibility to promote health and improve quality of life (2). Health promotion is highlighted in Family Health Strategy (FHS) actions, which compose primary health care according to the Unified Health System (SUS) (3), and important for the National Oral Health Policy, which guides the care model focused on prevention rather than curative care (4).

In this perspective, oral health promotion (OHP) and prevention actions are important to reduce the prevalence of oral diseases and costs of dental treatments in health institutions (5). Oral diseases are a global public health issue that leads to economic problems and impairs quality of life. This issue also reflects socioeconomic inequalities, lack of access to health services, and lack of incentive for prevention and treatment, particularly in low- and middle-income countries (6).

Contextual characteristics at municipal level may influence some oral health outcomes (7-9). For example, Brazilian adults from places with low Human Development Index (HDI) and high Gini index (more unequal) are more likely to suffer the impact of oral conditions on quality of life (7). In contrast, populations with high oral health coverage in primary care (8) and better HDI-Longevity (9) present greater use of dental services.

In this context, knowledge regarding relationships between social and economic contexts according to municipal inequalities, the scope of basic health services, and OHP procedures are important since gaps involving OHP and social determinants still exist. Health demands, availability of services, social environment, and geographic factors may also affect equity in oral health (10). Therefore, these factors are essential to achieving equity and universal OHP offer and improving health conditions of most vulnerable populations.

Despite the guidelines of the Brazilian National Oral Health Policy mentioning that Oral Health Promotion is “inserted in a broad concept of health that transcends the merely technical dimension of the dental sector”, in a limited way, the Brazilian Ministry of Health makes available among the promotion and prevention actions in oral health, in the Management System of the Table of Procedures, only those procedures considered eminently dental. For this reason, this analysis of national production focused on the procedures that SUS codifies in OHP, which represent only a portion of the actions inserted in the broad concept of promoting health.

Based on different realities of Brazilian municipalities, we hypothesized that sociodemographic and FHS coverage indicators would influence OHP procedures. Given the low number of national studies in this context and considering the perspective of collective and clinical OHP procedures, this study aimed to answer the following question: What are the associations between OHP procedures and sociodemographic conditions and FHS coverage in Brazilian municipalities?

## Material and Methods

-Study design

This cross-sectional study used a quantitative methodological approach based on secondary descriptive and analytical data. The study included municipalities of all Brazilian federal units and macro-regions that sent material from oral health teams via the Health Information System for Primary Care.

-Outcomes and covariates

The rates of two OHP outcomes (clinical and collective procedures) from 2019 were included. The rate of clinical procedures in each municipality was calculated by summing all procedures of this category and dividing by the total population of the municipality. A similar procedure was used to obtain the rate of collective procedures.

The following sociodemographic variables of municipalities were collected: Gini index, HDI, per capita income, rate of head-of-household mothers, rate of extreme poverty, rate of people vulnerable to poverty, and illiteracy rate. Gini Index measures income inequality (0 corresponds to absolute equality and 1 to absolute inequality) and indicates differences between the poorest and richest people of each location. For this study, Gini index was dichotomized into ≤ 0.50 and > 0.50 (11). Population size was categorized according to number of inhabitants: < 5,001; from 5,001 to 10,000; from 10,001 to 50,000; from 50,001 to 100,000; from 100,001 to 500,000; and > 500,000 (12). HDI was ranged from 0 to 1 (the higher the value, the better the social conditions). Values were categorized as low (≤ 0.50); medium (0.51 to 0.79); and high (> 0.80) (12).

The rate of head-of-household mothers was calculated by the ratio between the number of females responsible for the household, who did not complete primary education, and had at least one child aged < 15 years living in the household and total number of female heads of household multiplied by 100. Per capita income was calculated as the ratio between the sum of income of all individuals residing in permanent private households and the total number of individuals. The rate of extreme poverty considered the proportion of extremely poor people (monthly per capita income of < R$70.00), whereas the rate of people vulnerable to poverty considered those with per capita income equal to or less than half minimum wage (minimum wage for the year 2010 was R$510.00). Last, illiteracy rate of the population aged > 15 years was assessed as the ratio between those aged > 15 years who could not read or write a simple note and the total number of people of this age group multiplied by 100.

FHS coverage was represented as the percentage of population covered by family health teams (FHST) and oral health teams (OHT), calculated as the number of teams implemented for every 3,000 people divided by the total population residing in the municipality and multiplied by 100. The FHST and OHT coverage rates considered in the analyses were for December 2019. The percentages of the population covered by FHS and oral health team (OHT) were dichotomized in ≤ 50% and >50% (13).

-Data collection 

OHP procedures per municipality were requested in the web portal of the Brazilian Ministry of Health (http://sigtap.datasus.gov.br/). Therefore, the description of procedures and respective codes were verified. Clinical OHP procedures listed were topical application of fluoride, evidence of dental biofilm, and cariostatic and dental sealant application per tooth. On the other hand, collective OHP procedures referred to actions conducted by the OHT for group of individuals outside clinical settings such as: topical application of fluoride gel, fluoride mouthwash, supervised tooth brushing, oral examination with epidemiological purposes, and educational activities and/or group guidance in primary care.

Sociodemographic and health coverage variables were obtained using public information systems. The former was obtained using the Atlas of Human Development in Brazil (http://atlasbrasil.org.br) and based on data from the last demographic census (2010) performed by the Brazilian Institute of Geography and Statistics (IBGE). Health coverage variables were obtained using the e-Gestor Primary Care Electronic Portal of the Ministry of Health (https://egestorab.saude.gov.br/index.xhtml).

-Data analysis

The first outcome, individual procedures, was dichotomized based on median (0.027) and interquartile range (0.050 – 0.803), maximum-minimum 0.000-3.107. In this analysis, the municipalities were distributed among those with higher or lower production (they perform procedures above or below the median).

For the second outcome, collective procedures, in which the median was equal to 0.000; interquartile range (0.021—0.065), maximum-minimum 0,000 - 4,341, the municipalities were distributed among those who performed or not some procedure.

Five sociodemographic covariates were dichotomized according to the median: rate of head-of-household mothers (< 48.27% and ≥48.27%), per capita income (< R$424.27 and ≥ R$424.27), rate of extreme poverty (< 8.26% and ≥ 8.26%), rate of vulnerability to poverty (< 48.27% and ≥ 48.27%), and illiteracy rate (≤ 14.69% and > 14.69%).

For each outcome, negative binomial regression models were used to associate the two outcomes (clinical and collective OHP procedures) with covariates and estimate unadjusted and adjusted prevalence ratios (PR), confidence intervals (95%CI), and *p-value*s. Initially, the unadjusted negative binomial regression model was used to assess the independent effects of each covariate. The adjusted negative binomial regression model included only covariates with *p-value*s of < 0.25. The final model considered associated covariates when *p* < 0.05. For the evaluation of goodness of fit of the final model, the ratio between residual deviance and degree of freedom and the chi-squared test of the residual deviance results was indicated (14). All analyzes were performed using the Statistical Program for Social Science, version 25.0 (SPSS for Windows, SPSS, Inc., Chicago, IL, USA).

## Results

From 5,570 Brazilian municipalities, 4,913 (88.2%) presented data regarding clinical and collective OHP procedures from 2019. We observed a predominance of municipalities with low-income inequality (n=2,683; 54.6%), small population size (10,001 to 50,000 inhabitants) (n=2,206; 44.9%), and medium HDI (n=4,831; 98.3%). Regarding coverage indicators, 94.7% (n=4,655) and 83.7% (n=4,110) of municipalities had more than 50% of the population assisted by FHST and OHT, respectively (Table 1).

**Table 1:**
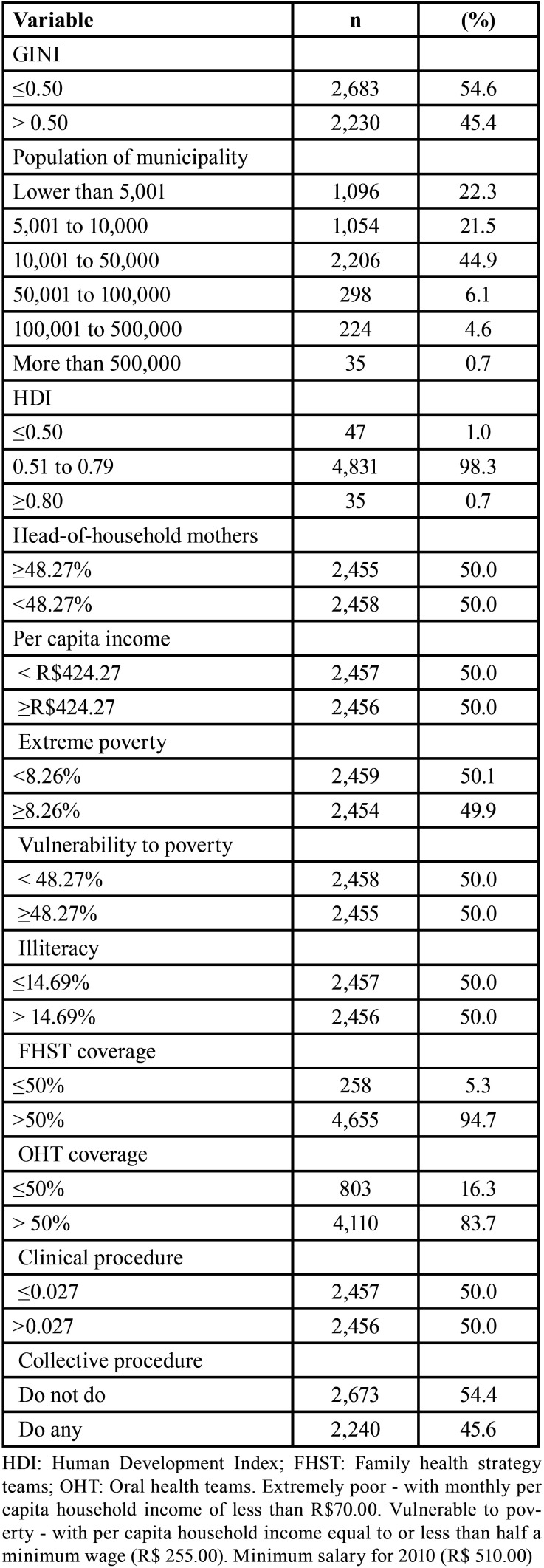
Distribution of municipalities according to sociodemographic variables and Family Health Strategy coverage, Brazil, 2019.

The final model indicated that municipalities with low Gini index had more frequency of performing clinical OHP procedures (PR=1.04, 95%CI 1.01 to 1.08) above the median. Those with high illiteracy rates had more frequency of performing the same procedures (PR=1.06, 95 %CI 1.00 to 1.13). Municipalities with 10,001 to 50,000 inhabitants (PR=1.07, 95%CI 1.02 to 1.12) and with 50,001 to 100,000 inhabitants (PR=1.21, 95%CI 1.12 to 1.30) had higher frequencies of clinical procedures than smallest municipalities. Municipalities with low vulnerability to poverty had less frequency of clinical procedures (PR=0.83, 95%CI 0.78 to 0.89). Finally, those with OHT coverage up to 50% of the population had less frequency of performing clinical procedures (PR=0.39, 95%CI 0.33 to 0.45) (Table 2).

**Table 2:**
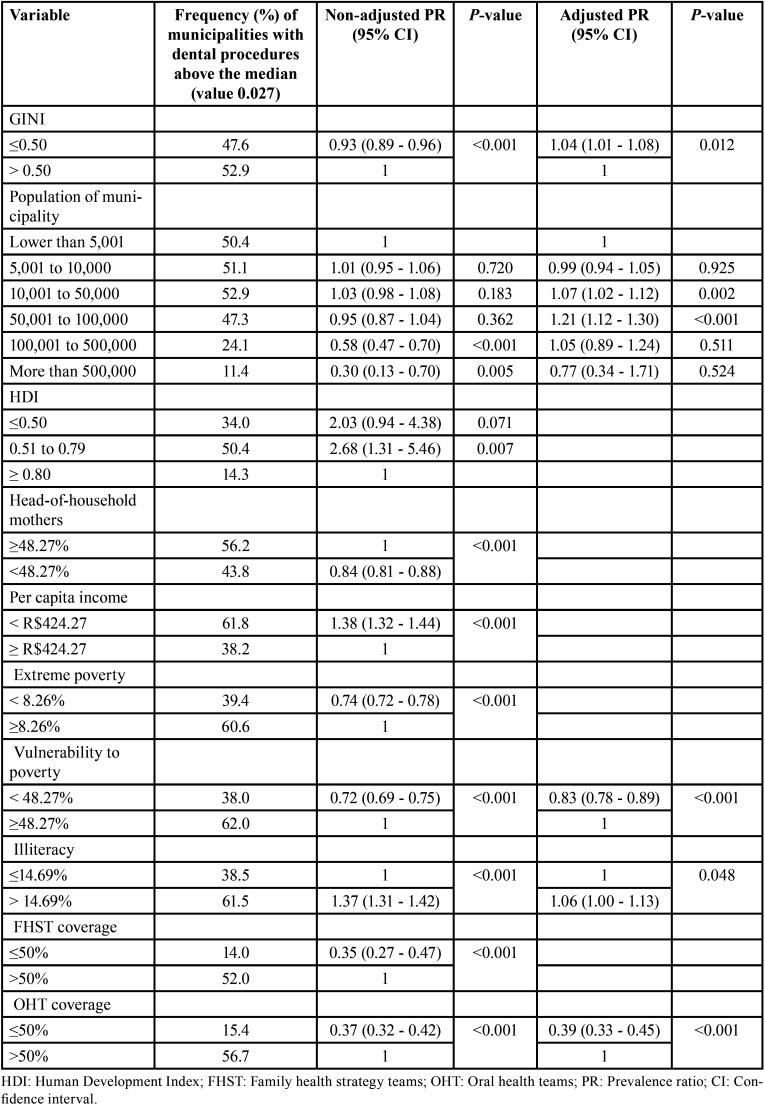
Factors associated with clinical health promotion procedures, Brazil, 2019.

Regarding collective OHP procedures, the final model demonstrated a low frequency of procedures among the least unequal municipalities (PR=0.91, 95%CI 0.87 to 0.95), among those with low (PR=0.53, 95%CI 0.35 to 0.80) and medium HDI (PR=0.79, 95%CI 0.71 to 0.87), and, finally, in municipalities with low per capita income (PR=0.84, 95%CI 0.81 to 0.88) (Table 3).

**Table 3:**
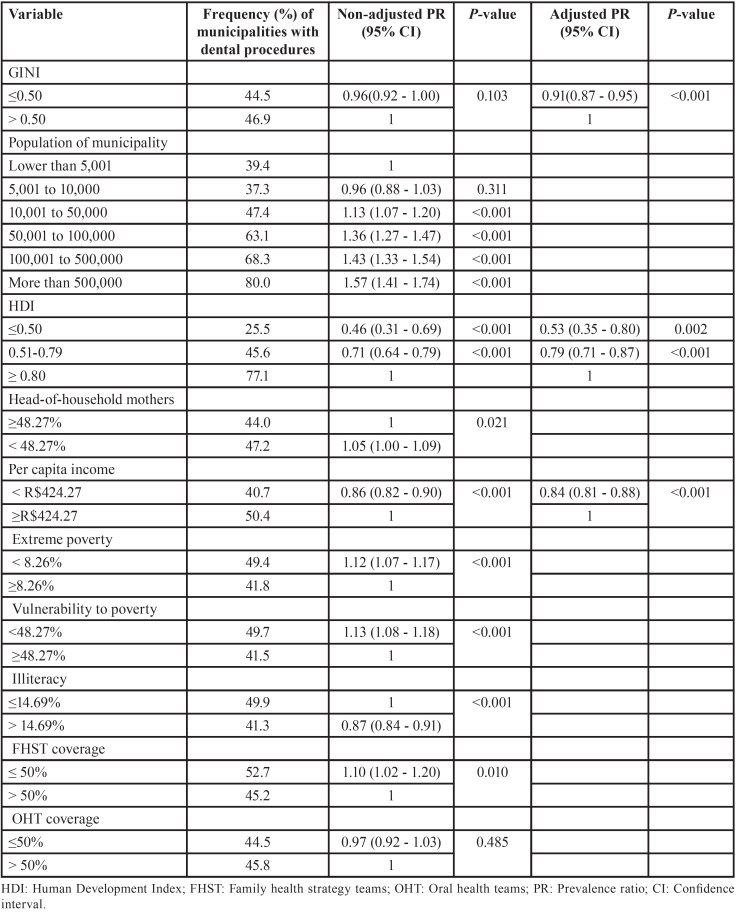
Factors associated with collective health promotion procedures, Brazil, 2019.

## Discussion

This study suggests that sociodemographic and OHT coverage influence the number of clinical and collective OHP procedures performed in Brazilian municipalities. We observed that municipalities that performed clinical procedures above the median presented low-income inequalities, high illiteracy rates, and medium-sized populations. Municipalities with greater vulnerability to poverty and low FHS coverage performed few clinical OHP procedures above the median. More collective procedures were found in municipalities with low GINI indices, low and medium HDI, and low per capita income.

Regarding income, the least unequal municipalities performed the highest number of clinical OHP procedures and the smallest number of collective procedures. Worldwide, income inequality is associated with poor oral health conditions. For example, low individual or family income is associated with oral cancer, prevalence and experience of dental caries, dental trauma, periodontal disease, and poor oral health-related quality of life (15). This indicates that most unequal populations are those with greatest need for OHP actions to promote equity in health.

Municipalities with highest illiteracy rates performed procedures above the median. This result is favorable since the lower the level of understanding of the population, the greater the need to use public dental services (8) and perform actions that lead to better health. According to IBGE criteria, illiterate people can not read and write even a simple note. This is worrying since the responsibility of knowledge sharing within the family is transferred to educated parents (16,17). Poor knowledge, attitudes, oral health practices, and low parental literacy are greatest predictors of dental caries in early childhood (18). In general, the chances of oral diseases are significantly higher in individuals with lower education (19,20).

The frequency of municipalities with procedures above the median was higher in medium-sized populations than those with < 5,000 inhabitants. A study on the conditions influencing the management of the local health system (categorized into favorable, regular, or unfavorable) revealed that 77% to 100% of large municipalities are in the favorable category, whereas only 10% to 17% of small municipalities are in the same condition (21). Given this finding, municipalization of health must be highlighted since it increases pressure on local governments to offer universal, resolute, and quality oral health care (22). Moreover, a great part of the health budget for municipalities comes from their resources, and they have little or no financial support from state health secretariats. Therefore, larger and more economically developed municipalities have more capacity to meet health demands due to greater availability of resources.

Municipalities with a small percentage of the population in the condition of vulnerability to poverty were associated with low frequency of clinical OHP procedures. Vulnerability to poverty reveals socioeconomic deficiencies of municipalities since worse socioeconomic conditions reflect oral health problems (6,7,15,19,20,23,24), and oral health status is an indicator of poverty (23). Therefore, reducing poverty may prevent illnesses and decrease hospitalizations and healthcare costs (24). Furthermore, OHP practices should be expanded to improve health of the population, while vulnerability to poverty should complement public policies to generate extensive and long-term structural social changes for Fighting and eradicating poverty.

According to the Ministry of Health, the OHT coverage indicator is limited because it only measures the existence of teams and not the work performed. Therefore, its analysis must be complemented with information regarding quantity and quality of care and procedures. In this context, we identified a low frequency of municipalities with low OHT coverage that performed clinical OHP procedures, reflecting the influence of the low offer and difficult access to basic dentistry services and reduced clinical oral health promotion actions. Therefore, universal oral health coverage with access to effective and quality services may minimize inequalities in OHP.

Regarding collective OHP practices, the low frequency of municipalities that conducted topical application of fluoride gel, fluoride mouthwash, supervised tooth brushing, oral examination for epidemiological purposes, and educational activities were associated with low HDI and low per capita income. In this context, we highlight the expansion of the offer of oral health services in the perspective of health promotion and considering universality, equity, integrality, and principles of SUS, mainly because worse living and health conditions reflect inequalities (15). For example, Pereira *et al*. (25) observed associations between the prevalence of dental caries in children aged 12 years and municipal HDI of Brazilian state capitals.

Additionally, the need to treat chronic oral diseases is high in low- and middle-income countries because costs may exceed available resources (6). For this reason, working effectively on OHP, in quantitative and qualitative terms, should be a priority for oral health managers.

This study has limitations regarding the cross-sectional design, and causal relationships cannot be determined. In addition, data regarding OHP procedures, such as oral hygiene recommendations, collective action for prevention of oral cancer, provisional sealing of dental cavity, and guidance on cleaning dental prostheses, were not sent by the Ministry of Health because they were not included in the SUS Table before September 2021.

The results of this study revealed inequities of practices in PSB among Brazilian municipalities. This points to challenges to be solved to ensure the assumptions of the National Oral Health Policy, within the scope of primary health care. The number of procedures seems to be associated with sociodemographic conditions and the coverage of ESB in the family health strategy, improvements related to the social dimension are as crucial as the expansion of health care.

Efforts must transcend the field of health in Brazil to reduce inequalities in OHP and improve contextual indicators of municipalities, territories, and populations by expanding the offer of public oral health services and changing economic and social policies.
